# Socioeconomic disparities in six-year incident dementia in a nationally representative cohort of U.S. older adults: an examination of financial resources

**DOI:** 10.1186/s12877-020-01553-4

**Published:** 2020-05-06

**Authors:** Laura J. Samuel, Sarah L. Szanton, Jennifer L. Wolff, Katherine A. Ornstein, Lauren J. Parker, Laura N. Gitlin

**Affiliations:** 1 Johns Hopkins University School of Nursing, 525 North Wolfe St., Rm 426, Baltimore, MD 21205 USA; 2 Johns Hopkins Bloomberg School of Public Health Department of Health Policy and Management, Baltimore, USA; 3 Icahn School of Medicine at Mount Sinai, Department of Geriatrics and Palliative Medicine and Institute for Translational Epidemiology, New York, USA; 4 Johns Hopkins Bloomberg School of Public Health Department of Health, Behavior and Society, New York, USA; 5 Drexel University College of Nursing and Health Professions, Philadelphia, USA

**Keywords:** Financial strain, Socioeconomic factors, Dementia, Health disparities, Education, Income, Aging

## Abstract

**Background:**

Less educational training is consistently associated with incident dementia among older adults, but associations between income and financial strain with incident dementia have not been well tested in national samples. This is an important gap because, like education, financial resources are potentially modifiable by policy change and strengthening the social safety net. This study tested whether financial resources (income and financial strain) predict six-year incident dementia independent of education and occupation.

**Methods:**

The National Health and Aging Trends Study is a prospective cohort study that recruited a nationally representative sample of U.S. Medicare beneficiaries aged ≥65 years. Incident dementia (2013 to 2018) was classified based on diagnosis, cognitive test scores or proxy-reported changes among participants dementia-free in 2012 (*n* = 3785). Baseline socioeconomic measures included income to poverty ratio (analyzed separately for those < 500% vs. ≥500% poverty threshold), financial strain, education and history of professional occupation. Discrete time survival analysis applied survey weights to account for study design and nonresponse. Coefficients were standardized to compare the strength of associations across the four socioeconomic measures.

**Results:**

Adjusting for socioeconomic measures, demographic characteristics, home ownership, retirement, chronic conditions, smoking, BMI and depressive symptoms, higher income (hazard OR = 0.84, 95% CI: 0.74, 0.95 among those < 500% poverty) and higher education (hOR = 0.73, 95% CI: 0.65, 0.83) were associated with lower odds, and financial strain with higher odds (hOR = 1.20, 95% CI: 1.09, 1.31), of incident dementia.

**Conclusion:**

Low income and greater financial strain predict incident dementia among older adults and associations are comparable to those of low education among U.S. older adults. Interventions to mitigate financial strain through improving access to economic opportunity and strengthening safety net programs and improving access to them in low income groups may complement other ongoing efforts to prevent dementia.

## Background

Dementia affects 47 million individuals globally [1]. Low-resourced populations tend to experience disproportionately higher dementia rates [2, 3] but associations between socioeconomic status and incident dementia are under-examined. Socioeconomic status is a multi-dimensional construct capturing both tangible and intangible resources that can prevent dementia via different intervening mechanisms [4]. As examples, higher educational training and greater occupational complexity are believed to contribute to greater cognitive reserve, which is an intangible resource that may prevent clinical signs of dementia despite pathological changes [5], whereas financial resources, such as income and financial strain, are more important for tangible resources such as nutrition and health care [4]. Determining which dimensions of socioeconomic status contribute to incident dementia could guide prevention and policies directed at lowering risks.

Not all socioeconomic measures have been examined with regard to incident dementia in population-based samples. Low education [3] and low occupational complexity [6] have both been associated with incident dementia in population-based studies. In fact, low education has been identified as a contributor to excess dementia incidence in the U.S. and globally [1, 7]. However, there is less data about whether income and financial strain predict incident dementia independent of education in national samples of older adults, despite two good reasons to investigate. First, income and financial strain have been consistently associated with health-related dementia risk factors, such as cardiovascular disease, smoking and depression [3, 8–10]. Secondly, there is some evidence linking income and financial strain with dementia independent of education, but results are not consistent across studies. Although income did not predict dementia-related mortality in a large sample of Norwegian men [11], lower income predicted memory decline in the U.S. nationally representative Health and Retirement Study [12], both lower income and financial strain predicted incident dementia in the Health ABC study, which sampled healthy black and white U.S. older adults [2], and fewer financial assets, which is related to income and financial strain, predicted incident dementia in a nationally representative sample of English older adults [13]. Also, it is important to test both income and financial strain, since income improves the quantity and quality of resources to prevent dementia and financial strain reflects whether the income is sufficient for an individual’s cost of living and financial commitments. Therefore, the current study tested the hypothesis that financial resources, including income and financial strain, predict incident dementia over 6 years independent of education and occupation among a nationally representative sample of adults aged 65 years and older.

## Methods

### Sample

**Fig. 1:**
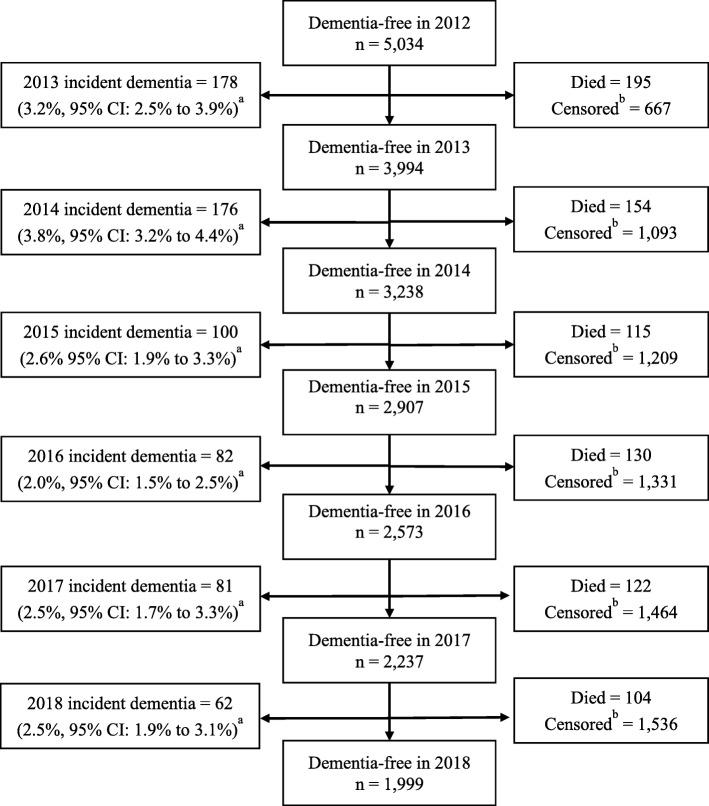
Flowchart of incident dementia, death and censoring at annual interviews, 2012 to 2018, among older adult participants of the National Health and Aging Trends Study aged 66 and older who were free of dementia in 2012. ^a^ One-year cumulative incidence. Sampling weights were used to represent the population of Medicare beneficiaries aged 66 years and older in 2012. ^b^ In this study, censoring was defined as missing dementia data from an annual interview. Censoring was treated as a repeatable event and counts represent cumulative censoring. A small number of participants returned for an annual interview after missing a prior interview, and any data collected after the return was included in analyses. FIML analyses included all available data available up to the point of death or permanent censoring

The National Health and Aging Trends Study (NHATS) is a cohort study of U.S. Medicare beneficiaries ages 65 and older that utilized stratified random sampling described elsewhere [14]. NHATS had a response rate of 71% at baseline in 2011. In-home interviews are conducted annually by trained interviewers. As financial strain is a key independent variable in this study and was first included in 2012, we evaluate incident dementia between 2013 and 2018 among participants who were dementia-free in 2012. Of the 7609 NHATS participants who completed the first interview in 2011, 6056 (96%) completed the 2012 interview and 5034 (84%) did not have dementia in 2012 and were included in this study. About 45% of the total sample (*n* = 2381) contributed less than 6 years of follow up data; 820 (13%) died and 1617 (33%) participants were otherwise missing from at least one annual interview (see Fig. 1). Participants who died or missed an interview were older (46% were aged ≥75 years vs. 39%), less likely to have a Bachelor’s degree (29% vs. 36%) or professional occupation (36% vs. 42%) and more likely to be black (8% vs. 7%) than white, but did not differ by Hispanic ethnicity, gender, financial strain or income level compared to participants with complete information across all 6 years. Differential nonresponse to the 2012 interview was addressed using 2012 sampling weights. Differential attrition between 2013 and 2018 was addressed using Full Information Maximum Likelihood. Full information Maximum Likelihood estimates model parameters using all available information without excluding participants or imputing values and produces unbiased estimates for data missing at random in adjusted models [15]. NHATS was approved by the Johns Hopkins Bloomberg School of Public Health IRB. These analyses were deemed exempt by the Johns Hopkins School of Medicine IRB. Participants provided informed consent.

### Primary outcome

Incident dementia was categorized based on a validated protocol for classifying “probable” dementia [16] as in prior work [17]. Self-responding participants completed five tests, described elsewhere [16] which evaluated three cognitive domains: memory, orientation, and executive function. Proxy respondents completed the AD8 Dementia Screening Interview, a validated eight-item screener for proxy respondents measuring memory, temporal orientation, judgment and function [18]. Participants were classified as having dementia if (1) the participant or a proxy reported that the participant had been told by a doctor that he/she has dementia or Alzheimer’s disease or (2) scores were ≥ 2 on the AD8 screener or (3) cognitive test scores were at or below 1.5 standard deviations below the mean for self-responding participants in at least two domains.

### Socioeconomic status

Measures of socioeconomic status included education, income, financial strain, and occupation based on participant or proxy responses. Educational training was ordinal (< High School, High School, some college, and ≥ Bachelor’s degree). Income was measured as income to poverty ratio to account for household size, calculated as the ratio of 2011 household income to the relevant 2011 US Census Bureau poverty threshold for individuals aged ≥65 years based on household size. Due to non-linear associations between the income to poverty ratio and the logit of incident dementia in this study, a piecewise spline term was created with a knot at the 500% poverty threshold. Therefore, the association between the income to poverty ratio and incident dementia was tested separately among those with incomes < 500% and among those ≥500% of the poverty threshold. The 500% poverty threshold was deemed most appropriate for the data based on examination of locally weighted smoothed regression plots (see Supplemental Fig. 1). Participants were classified as having financial strain in 2012 if they lacked money to pay the rent/mortgage, utility bills or medical/prescription bills in the past year or skipped any meals because there was not enough money to buy food in the past month. Professional occupation refers to self-reported longest occupation held using the US Census classification for the professional occupational category (i.e. management/professional occupation vs. all other occupations including service, sales/office, construction/farming, production, and homemaker).

### Background and health variables

Additional data used for these analyses include baseline (2011) age, gender, and race/ethnicity, [black, other race, Hispanic and white (referent)]. To capture qualitative differences in financial resources, we adjusted for 2012 retirement status (no/yes) and home ownership [not a homeowner (referent), paying mortgage, mortgage fully paid]. We also accounted for the following 2012 health-related dementia risk factors that may partially account for associations between socioeconomic status and incident dementia: pack years of cigarette smoking, body mass index (BMI), depressive symptoms, and diagnosis for heart attack/heart disease, high blood pressure, diabetes or previous stroke. BMI was calculated using reported height and weight. Pack years of smoking was calculated based whether the participant ever or currently smoked, the age he/she started and quit smoking, and the usual number of daily cigarettes. Presence of depressive symptoms was classified based on PHQ-2 score > 3 [19].

### Statistical approach

In NHATS, income data was missing for 31% of participants and was imputed in a process described in detail elsewhere [20]. All other variables for these analyses had ≤5% missing data. Bivariate standardized coefficients were used to evaluate the degree to which different socioeconomic measures capture different dimensions of socioeconomic status and to describe the inter-relationships between the four measures. Discrete time survival models, which use logistic regression [21], tested hypothesized associations between socioeconomic measures and incident dementia. Coefficients were standardized to compare the strength of associations across the four socioeconomic measures, which have different units of measurement. So, the hazard odds ratios (hOR) estimates the association between incident dementia with each standard deviation unit change in the socioeconomic measure. Piecewise regression was used to estimate the association between income to poverty ratio and incident dementia among participants with incomes < 500% poverty threshold, and separately, among participants with incomes ≥500% poverty. Model 1 included income to poverty ratio, educational training, professional occupation, financial strain, retirement, home ownership, age, gender, and race/ethnicity. Model 2 additionally adjusted for health-related dementia risk factors that may partially account for socioeconomic disparities in incident dementia, including heart disease, high blood pressure, diabetes or previous stroke, pack years of cigarette smoking, BMI and depressive symptoms. All analyses were conducted in Mplus version 7.3. Sample weights were used to account for sampling design and nonresponse by 2012.

Four different planned sensitivity analyses were conducted. First, due to the high correlation between financial strain and income, additional analyses excluded first financial strain, and then income, separately in two regression models. Second, to account for homemakers in this cohort whose non-professional status may not reflect their socioeconomic status, an interaction between gender and professional occupation was tested and separate models excluded homemakers. Third, analyses to address differential attrition excluded participants who did not provide complete data through 2018 and applied 2018 weights to draw inferences back to the target population. Finally, financial strain, which was the only socioeconomic measure with repeated values, was modeled as annually-variant using data from 2012 to 2017.

## Results

The six-year cumulative incidence of dementia was approximately 10.5% (Table 1), which extrapolates to 3,100,530 U.S. older adults transitioning to dementia over 6 years. Annual incidence rates ranged from 2.0% (95% CI: 1.5 to 2.5%) to 3.8% (95% CI: 3.2 to 4.4%) in the weighted sample (Fig. 1). Participants free of dementia in 2012 who were included in the analytic sample were typically < 75 years old (58%), female (56%), retired (51%), homeowners (55%) and white (84, 8% were black, 6% Hispanic, and 3% were other racial or ethnic groups). Characteristics according to dementia status by study completion (2018) are summarized in Table 1. Older adults who developed dementia (*n* = 679) were more likely to report financial strain at baseline, to have less than high school education, and were less likely to have worked mainly in a professional occupation than those who remained free of dementia in 2018 (*n* = 4355). The mean income to poverty ratio did not differ by 2018 dementia status in the total sample, but among a subset of participants with incomes < 500% poverty, those with incident dementia had a lower average income to poverty ratio than their peers who remained free of dementia in 2018. Those with incident dementia were older and more likely to be female, black or Hispanic than white, more likely to report diabetes, a history of stroke and experience depressive symptoms.

**Table 1:** Baseline socioeconomic, demographic and health-related dementia risk factor characteristics according to dementia status by 2018 among those without dementia in 2012, National Health and Aging Trends Study (*n* = 5034)

	No dementia by 2018 *n* = 4355 (89.5%)	Dementia by 2018 *n* = 679 (10.5%)	*p* value
Mean income to poverty ratio (mean income)	4.97 ($66,020)	3.23 ($43,524)	0.211
Among participants < 500% poverty (*n* = 2362, 46%)	2.15	1.74	< 0.001
Among participants ≥500% poverty (*n* = 2672, 54%)	13.36	11.70	0.876
Financial strain (%)	252 (5)	79 (11)	< 0.001
Education (%)			< 0.001
< High school	840 (16)	234 (31)	
High school	1205 (28)	186 (29)	
Some college	1161 (28)	140 (21)	
≥ Bachelors	1120 (29)	115 (18)	
Professional occupation^a^ (%)	1626 (40)	204 (33)	0.003
Own home (%)			0.126
Not homeowner	897 (19)	174 (26)	
Paying mortgage	978 (26)	109 (18)	
Mortgage fully paid	2266 (55)	340 (56)	
Retired (%)	2305 (50)	415 (62)	< 0.001
Age (%)			< 0.001
65–69 years	1036 (34)	57 (12)	
70–74 years	1065 (28)	101 (19)	
75–79 years	905 (19)	144 (23)	
80–84 years	772 (12)	180 (24)	
85–89 years	390 (6)	113 (14)	
≥ 90 years	187 (2)	84 (7)	
Gender (%)			0.001
Male	1883 (44)	256 (38)	
Female	2472 (56)	423 (62)	
Race/ethnicity (%)			
White	3185 (85)	431 (76)	(ref.)
Black	844 (7)	169 (10)	0.004
Hispanic	188 (5)	56 (10)	0.001
Other	104 (3)	17 (3)	0.598
Mean BMI	27.77	26.97	0.006
Heart disease (%)	922 (20)	143 (21)	0.355
High blood pressure (%)	3011 (65)	486 (70)	0.081
Diabetes %	1102 (23)	195 (29)	0.016
History of Stroke (%)	71 (1)	24 (4)	0.001
Mean pack years smoking	15.62	15.71	0.941
Ever smoked (%)	2252 (53)	334 (52)	0.493
Depressive symptoms (%)	490 (10)	113 (17)	< 0.001

Note: 2012 sampling weights were used to represent the population of Medicare beneficiaries aged 66 years and older

^a^ Classified as professional vs. all other occupational categories (i.e. service, sales/office, construction/farming, production, and homemaker) based on U.S. Census and longest held occupation

Bivariate standardized coefficients between the socioeconomic measures revealed key differences across measures. Financial strain was strongly inversely associated with income (B = − 0.974, *p* < 0.001), but had modest associations with education (B = − 0.273, p < 0.001) and professional occupation (B = − 0.165, p < 0.001). Education was moderately positively associated with both income (B = 0.713, *p* = 0.004) and professional occupation (B = 0.594, p < 0.001). Income was not associated with professional occupation (B = 0.202, *p* = 0.490).

**Fig. 2:**
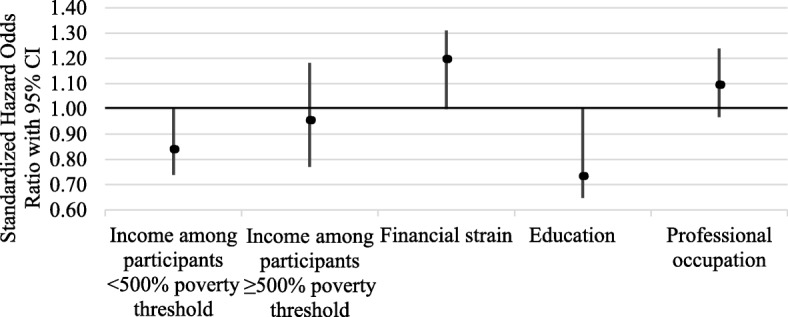
Standardized hazard odds ratios and 95% CI’s of associations between baseline measures of socioeconomic status and six-year incident dementia from discrete time survival models, adjusting for demographic characteristics, socioeconomic status and health-related dementia risk factors (Model 2), National Health and Aging Trends Study (2013–2018, *n* = 3785). Sampling weights were used to represent the population of Medicare beneficiaries aged 66 years and older in 2012. Associations between income and incident dementia were estimated using piecewise linear regression allowing the slope to differ for participants < 500% poverty threshold and participants ≥500% poverty threshold. Education was measured as < High School, High School, some college, and ≥ Bachelor’s degree

Adjusting for age, race/ethnicity, gender, home ownership, retirement, and all socioeconomic measures, three socioeconomic measures predicted incident dementia over 6 years (Table 2, Model 1). Financial strain predicted higher odds, while higher education and higher income (among those with incomes < 500% poverty threshold) predicted lower odds of incident dementia. Effect sizes were similar across these three measures; hazard odds ratio estimated between 19 and 28% higher or lower odds of incident dementia. Professional occupation and higher income among those with incomes exceeding the 500% threshold did not predict incident dementia. Additional adjustment for health-related dementia risk factors did not alter inferences (Table 2, Model 2) and hazard odds ratios were similar to those in Model 1. As shown in Fig. 2, hazard odds ratios were fairly similar for financial strain, education, and higher income among participants with incomes < 500% poverty. Four separate sensitivity analyses were conducted as described in statistical methods and inferences were unchanged for each (results not shown).

**Table 2:** Associations between measures of socioeconomic status and subsequent incident dementia (2013–2018) among National Health and Aging Trends Study participants

	Model 1 (***n*** = 3917)			Model 2 (n = 3785)		
	hazard OR	(95% CI)		hazard OR	(95% CI)	
Income to poverty ratio among participants < 500% poverty^a^	0.81	(0.71	0.92)	0.84	(0.74	0.95)
Income to poverty ratio among participants ≥500% poverty^a^	0.99	(0.88	1.12)	0.96	(0.77	1.18)
Financial strain	1.21	(1.10	1.32)	1.20	(1.09	1.31)
Education	0.72	(0.63	0.81)	0.73	(0.65	0.83)
Professional occupation	1.11	(0.98	1.26)	1.10	(0.97	1.24)
Age	2.03	(1.87	2.22)	1.93	(1.76	2.12)
Race/ethnicity						
White (ref.)						
Black race	1.10	(1.01	1.19)	1.09	(1.00	1.18)
Hispanic ethnicity	1.18	(1.08	1.28)	1.18	(1.08	1.28)
Other race/ethnicity	1.07	(0.97	1.19)	1.05	(0.94	1.17)
Female gender	0.98	(0.89	1.08)	1.02	(0.94	1.12)
Own home	0.98	(0.88	1.09)	1.00	(0.91	1.11)
Retired	1.16	(1.04	1.29)	1.17	(1.05	1.30)
Heart disease				1.01	(0.91	1.11)
High blood pressure				0.97	(0.87	1.08)
Diabetes				1.13	(1.01	1.27)
Stroke				1.10	(1.01	1.19)
Pack-years smoking				1.13	(1.03	1.22)
BMI				0.87	(0.77	0.98)
Depressive symptoms				1.12	(1.01	1.24)

Note: Annual classification of dementia is based on NHATS protocol for classifying ‘probable’ dementia based on (1) cognitive test scores among self-responding participants or (2) AD8 screener scores ≥2 among proxy-respondents or (3) report of physician diagnosis of dementia. Analyses restricted to individuals who were classified as dementia-free in 2012. Standardized coefficients were estimated from discrete survival analysis models in Mplus using full information maximum likelihood. Sampling weights were used to represent the population of Medicare beneficiaries aged 66 years and older in 2012. Model 1 adjusted for 2011 age, gender, race/ethnicity, income to poverty ratio, educational training, whether the participant had mainly a professional occupation, and 2012 financial strain, retirement and home ownership. Model 2 additionally adjusted for 2012 cardiovascular health characteristics, including history of heart attack or other heart disease, high blood pressure, diabetes or previous stroke, pack years of cigarette smoking and BMI, and depressive symptoms

^a^ Associations between income to poverty ratio and incident dementia were estimated using piecewise linear regression. So, the slope was allowed to differ for participants < 500% poverty threshold and participants ≥500% poverty threshold

## Discussion

This is the first nationally representative study of older adults showing that lower income and financial strain predict incident dementia and have associations distinct from low education but comparable in size. This study builds on prior studies linking income and financial strain with cognitive changes in older adults [2, 12]. Together, these results suggest that dimensions of socioeconomic status beyond education place individuals at risk with regard to dementia. These results call for greater attention to the role of financial resources and the lack thereof as predictors of dementia risk in older adults.

Why do lack of financial resources place older adults at increased risk for dementia? In this study, three components of socioeconomic status - financial strain, income and education - were independently associated with incident dementia. These results are consistent with the conceptual understanding of socioeconomic status as a multi-dimensional concept, each contributing to incident dementia via different intervening mechanisms [4]. Less cognitive reserve likely represents a key mechanism linking low education with incident dementia [5], but may not explain effects of income and financial strain noted in this study. Income and financial strain were not strongly correlated with education in this study, suggesting that they capture a different dimension of socioeconomic status. Both income and financial strain capture financial resources that can promote health. Despite the strong correlation between them, both measures independently predicted incident dementia, suggesting that both financial resources are important predictors of incident dementia. These results add incrementally to the other modifiable risks identified in *The Lancet* Commission on Dementia Report, which, if addressed, could further reduce population dementia risk beyond the one-third reduction proposed in that report [1].

These results add to prior literature linking financial resources with aging-related outcomes; low income and financial strain have predicted earlier mortality [22, 23] and disability [24]. Notably, in this and a prior study [22], income was most strongly associated with health outcomes among those living at or near the poverty threshold. Together, these results warrant further attention to explore intervening mechanisms between financial resources and aging-related outcomes for older adults and suggest that the lowest income individuals should be targeted for prevention efforts.

Notably, three socioeconomic measures remained significant in regression models that adjusted for health-related dementia risk factors, suggesting that socioeconomic associations with dementia risk cannot be attributed to late life chronic disease, smoking history and depression. Although it is possible that the physiologic mechanisms linking chronic disease to dementia risk occur prior to older ages [25, 26], it is also possible that current socioeconomic status confers additional dementia risk via pathways unrelated to chronic disease burden. The theory of fundamental causes proposes that low socioeconomic status causes disease through multiple mechanisms [4]. One key mechanism may include physiologic dysregulation due to relatively more exposure to negative life events and adverse environmental conditions that provoke stress response mechanisms in low socioeconomic status groups. Physiologic dysregulation predicts earlier disability, mortality and cognitive decline [27]. A second mechanism may include less attention to cognitive tasks because of relatively more attention consumed by financial worries [28]. Regardless of the mechanisms, these results suggest that interventions that solely target older adult health-related risk factors will likely not prevent dementia for low socioeconomic status groups. One study showed that recent U.S. declines in dementia prevalence are likely due to age- and education-shifts in the population [17], suggesting that efforts to address socioeconomic exposures may reduce the global dementia burden. Together, these studies suggest that interventions aimed at preventing dementia should move upstream in the causal chain by addressing socioeconomic conditions.

Financial resources may be more modifiable than other socioeconomic dimensions among older adults. Despite the empirical link between education and dementia, education typically captures early life opportunities, whereas financial resources capture an older adult’s current resources. Similarly, while education is not typically acquired in midlife or late life when individuals experience the greatest risk for dementia, financial strain may be modified in late life by improving access to programs providing financial assistance for daily necessities. Financial strain suggests two possible intervention targets –increasing income or decreasing expenses such as medication costs. As examples, one study found that a randomly distributed influx of income improved attention and fluid intelligence scores for low-income adults [28], suggesting that increased financial resources may improve cognitive ability. Also, financial strain is less strongly associated with lower well-being in European countries with relatively more generous welfare programs [29]. As U.S. examples, the implementation of Medicare Part D is associated with lower cost-related medication non-adherence [30] and participation in the Supplemental Nutrition Assistance Program (SNAP) improves an individual’s ability to afford food [31], reduces health care utilization [32, 33], and reduced cost-related medication non-adherence [34]. Despite documented benefit from SNAP, only 42% of eligible older adults actually participate in SNAP [35] and the complex enrollment process for such programs may pose a barrier for older adults. Rigorous evaluation of public benefits and other programs may improve our understanding of how to reduce financial strain for vulnerable older adults.

### Strengths and limitations

Reverse causation is a limitation if pre-clinical cognitive decline influenced financial resources prior to study initiation. These results from an older adult cohort may have been affected by survival bias, since individuals of low socioeconomic status may have developed dementia or died before age 65. The use of proxy respondents in this study may have increased risk of measurement error for socioeconomic exposures, although the inclusion of participants with proxy respondents likely improved the representativeness of the sample. Older adults may rely on accumulated financial assets other than home ownership that were not captured in this study. Strengths of this study include a nationally representative cohort of US adults over age 65 years, use of validated cognitive measures and multiple socioeconomic measures.

## Conclusions

Dementia is burdensome, costly and feared. Given the financial burdens of dementia on individuals and families [36], attending to financial strain as a risk factor is important. These results contribute to prior studies by showing in a national representative sample that individuals with the fewest financial resources are the most likely to acquire dementia. Prior work has identified multiple modifiable risk factors for dementia [1] and this study builds on those findings by identifying financial resources as a potentially modifiable risk factor. Greater attention is needed to improve financial support for dementia-related prevention and care for low-income and financially strained older adults.

## Supplementary information

**Additional file 1: Figure S1.** Logit-smoothed Locally Weighted Scatterplot Smoothing (LOWESS) graph, showing non-linear relationship between logit of five-year cumulative incident dementia (between 2013 and 2018) and the 2011 income to poverty ratio among National Health and Aging Trends Study participants free of dementia in 2012, separately for each of the five multiply imputed datasets among participants with income to poverty ratios < 100 (i.e. < 10,000% poverty) (*n* = 5023 to 5025 across the five datasets).

## Data Availability

The NHATS data analyzed in the current study are available for research purposes at www.nhats.org.

## Abbreviations

U.S.: United States
NHATS: National Health and Aging Trends Study
BMI: body mass index
