# Supplementary material for: Socioeconomic disparities in six-year incident dementia in a nationally representative cohort of U.S. older adults: an examination of financial resources
Source: BMC Geriatr. 2020 May 6;20:156. doi: 10.1186/s12877-020-01553-4 (PMC7201761; doi:10.1186/s12877-020-01553-4)
Supplement: Supplementary file 1 — Additional file 1: Figure S1. Logit-smoothed Locally Weighted Scatterplot Smoothing (LOWESS) graph, showing non-linear relationship between logit of five-year cumulative incident dementia (between 2013 and 2018) and the 2011 income to poverty ratio among National Health and Aging Trends Study participants free of dementia in 2012, separately for each of the five multiply imputed datasets among participants with income to poverty ratios < 100 (i.e. < 10,000% poverty) (n = 5023 to 5025 across the five datasets). [file 12877_2020_1553_MOESM1_ESM.docx]

Supplemental Figure 1

Logit-smoothed Locally Weighted Scatterplot Smoothing (LOWESS) graph, showing non-linear relationship between logit of five-year cumulative incident dementia (between 2013 and 2018) and the 2011 income to poverty ratio among National Health and Aging Trends Study participants free of dementia in 2012, separately for each of the five multiply imputed datasets among participants with income to poverty ratios <100 (i.e. <10,000% poverty) (n=5,023 to 5,025 across the five datasets).
